# IgG1-b12–HIV-gp120 Interface in Solution: A Computational Study

**DOI:** 10.1021/acs.jcim.1c01143

**Published:** 2021-12-31

**Authors:** Didac Martí, Carlos Alemán, Jon Ainsley, Oscar Ahumada, Juan Torras

**Affiliations:** † Department of Chemical Engineering (EEBE), Universitat Politècnica de Catalunya, C/Eduard Maristany 10-14, Ed I2, 08019 Barcelona, Spain; ‡ Barcelona Research Center for Multiscale Science and Engineering, Universitat Politècnica de Catalunya, C/Eduard Maristany 10-14, 08019 Barcelona, Spain; § Institute for Bioengineering of Catalonia (IBEC), The Barcelona Institute of Science and Technology, Baldiri Reixac 10-12, 08028 Barcelona, Spain; ∥ Evotec Campus Curie, 195 Rte d’Espagne, 31100 Toulouse, Occitanie, France; ⊥ Mecwins S.L., Parque Científico de Madrid PTM, C/Santiago Grisolía 2, Tres Cantos, Madrid 28760, Spain

## Abstract

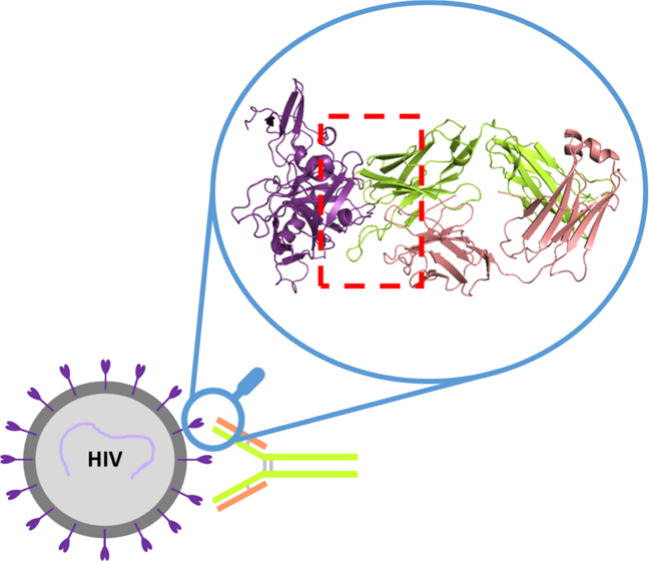

The use of broadly neutralizing antibodies against human immunodeficiency virus type 1 (HIV-1) has been shown to be a promising therapeutic modality in the prevention of HIV infection. Understanding the b12–gp120 binding mechanism under physiological conditions may assist the development of more broadly effective antibodies. In this work, the main conformations and interactions between the receptor-binding domain (RBD) of spike glycoprotein gp120 of HIV-1 and the IgG1-b12 mAb are studied. Accelerated molecular dynamics (aMD) and ab initio hybrid molecular dynamics have been combined to determine the most persistent interactions between the most populated conformations of the antibody–antigen complex under physiological conditions. The results show the most persistent receptor-binding mapping in the conformations of the antibody–antigen interface in solution. The binding-free-energy decomposition reveals a small enhancement in the contribution played by the CDR-H3 region to the b12–gp120 interface compared to the crystal structure.

## Introduction

The human immunodeficiency virus (HIV) is the causative agent of acquired immune deficiency syndrome (AIDS);^1^ it is estimated that 37.9 million people were living with HIV in 2018. Thankfully, the overall mortality in those affected by HIV was substantially reduced when combined antiretroviral therapy (ART) was introduced.^2^ It is estimated that ART halved the average mortality rate in HIV-infected individuals.^3^ In fact, people infected with HIV who adhere to ART can expect to live a near-normal life span.^4^ However, the development of an effective vaccine is a true challenge for medical science. Due to its high mutability and variability, HIV can evade the adaptive immune system; understanding the underlying physical mechanisms of immune evasion is therefore vital for development of an effective vaccine.^5,6^ Similarly, an in-depth understanding of the binding mechanism of broadly neutralizing HIV antibodies would aid the development of new or modified monoclonal antibodies (mAbs) to fight HIV,^7^ as well as the development of novel immunosensors with lower production costs and earlier detection windows.^8,9^

Host cell infection by the primate immunodeficiency viruses primarily occurs through the binding of the viral gp120 envelope glycoprotein to the host’s CD4 glycoprotein.^10^ Considering this entry point, researchers focused on developing a vaccine that could elicit antibodies that bind to the viral surface-exposed envelope glycoprotein (Env), and thus block the initial stage of infection of the host cells.^6^ Env is a heterodimer made of a transmembrane glycoprotein (gp41) and a surface glycoprotein (gp120), which together form a mushroom-shaped structure with the three gp41 components located at the base of the gp120 trimer.^11^ From a structural point of view and following the nomenclature introduced by Kwong et al.,^10^ the gp120 chain consists of two main domains (inner and outer domains) with several loops emanating from them. More specifically, the inner domain presents the V1/V2 loop at the distal end, forming the bridging sheet between both inner and outer domains. This bridging sheet is well known to be involved in the spatially separated interactions of gp120 with both CD4 and the 17b antibody, together with the strand β15 and helix α3, which are also important actors in the CD4-binding event. More specifically, the binding pocket where CD4 is bound consists of a depression located at the interface between the outer domain and the inner domain and the bridging sheet of gp120. This interaction involves about 802 Å^2^ of the gp120 surface.^10^ The proximal end of the outer domain includes the variable loops named V4, V5, *LD*, and *LE*. These variable loops (V1–V4) have been previously proposed to be all generally located in solvent-accessible regions.^12^ Recently, the interaction between the V1/V2 domain with the light chain (L) of the b12 antibody was studied using computational modeling.^13^

Few human monoclonal antibodies have been identified with efficient neutralization and the ability to protect against the viral charge in vivo.^14^ Among them, antibody b12 is the one that has been shown to be able to effectively neutralize a broad spectrum of primary isolates of HIV-1.^15^ This antibody recognizes a highly conserved epitope with an important coincidence with the CD4-binding region of gp120. Specifically, b12 neutralizes about 75% of clade B primary viruses and a similar or lesser ratio of other clades. Indeed, due to its potency and broad specificity, its epitope on gp120 has been noted as a particularly effective target for vaccine design.^14^

IgG1-b12 is unusual compared to other IgG1 antibodies since only the heavy chain directly interacts with gp120, while the light chain is not at all directly involved in such protein–protein interactions.^16^ The majority of the interactions between the complementarity-determining regions (CDR) of the heavy chain of IgG1 and the outer domain of gp120 take place on the interacting surface of the CDR region, which is composed of three loops (i.e., CDR-H1 to CDR-H3). These CDR loops of the b12 antibody bind to and bury the CD4-binding loop of the gp120 envelope protein, thus preventing the protein’s, and therefore the virus’s ability to bind to the CD4 receptors of the host lymphocytes, thus preventing their infection.^14,17^

**Figure 1:**
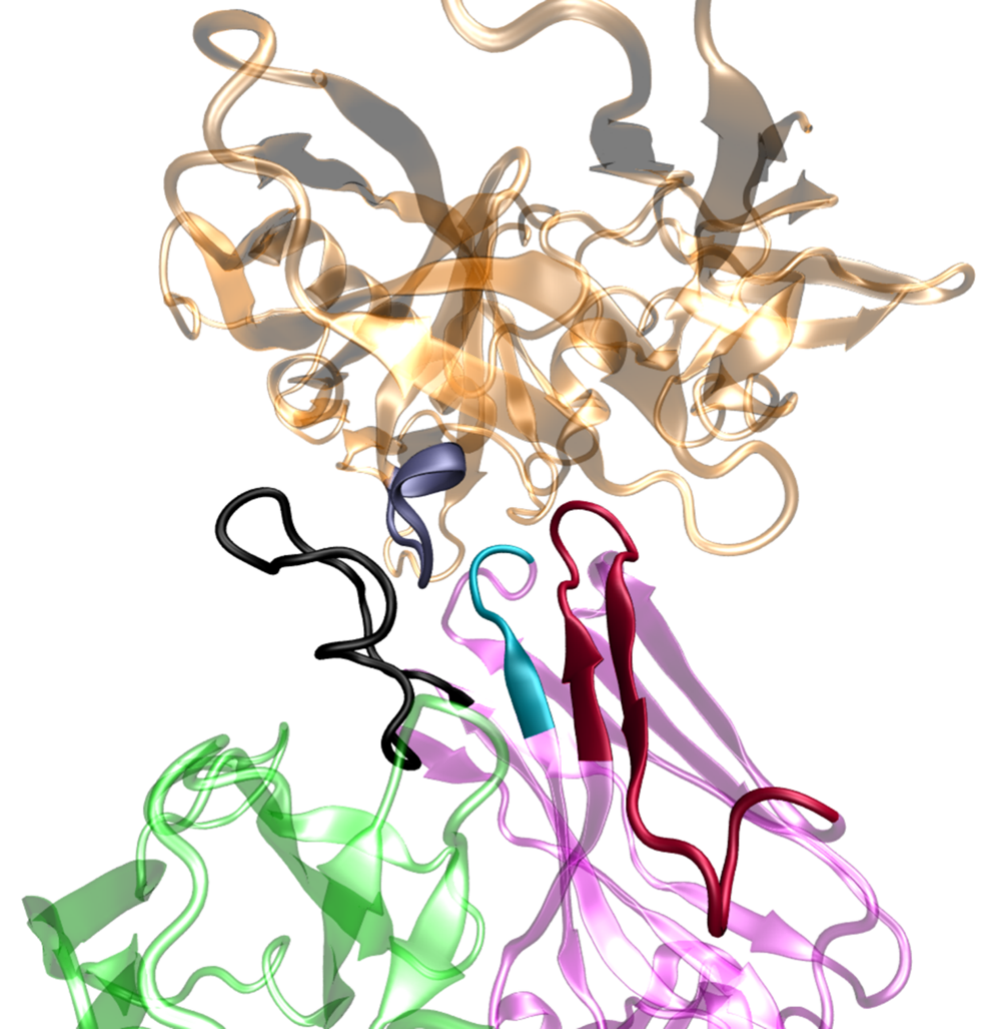
Model of the antibody–antigen complex between the IgG1’s Fab domain and gp120 protein of the HIV spike is detailed. The antigen (gp120 protein in orange), the heavy chain (pink), and the light chain (green) of the Fab domain are shown. Also, the CD4-binding loops of antigen (in ice blue), CDR-H1 (cyan), CDR-H2 (red), and CDR-H3 (black) are highlighted in the figure.

The b12 antibody’s CDR-H3 loop consists of 17 amino acids with a Trp100 residue at its extreme that extends up to 15 Å beyond the antigen-binding surface. All known gp120 binding antibodies have a similar length of the H3 loop.^14^ It has been shown with docking techniques that the surfaces of the IgG1-b12 antibody and the gp120 protein are complementary, where the H3 loop penetrates inside the pocket of the gp120 Phe43 together with other less significant interactions with other CDRs such as H1 and H2.^14^ However, crystallographic structures of IgG1 should only be considered as low-energy snapshots of the set of conformations available in solution. In this work, we will explore this set of structures in conditions close to the physiological ones in solution to observe the variations that may present the different interactions of the b12–gp120 complex (Figure 1). An extended study of different accessible conformations using accelerated molecular dynamics (aMD) and a classical force field is conducted. Selected snapshots from the most populated minima will be relaxed with ab initio quantum mechanics using a quantum mechanics/molecular mechanics molecular dynamics (QM/MM MD) approach to refine the chemical interactions on the interface in solution.

Knowledge of the role that other possible conformations may play in stabilizing the b12–gp120 interface in solution will provide a deep insight into the most probable interactions of the complex structures. These insights will facilitate the design of novel HIV vaccines by emulating an activity similar to or better than that exhibited by the b12 antibody.

## Methods

### b12–gp120 Protein Complex Model

The 2.3 Å resolution structure of the Fab region of the IgG1-b12 antibody complexed with the HIV CD4-binding domain of gp120 was taken from the protein data bank (pdb code 2NY7).^16^ The missing loop regions of gp120, which are distant from the b12–gp120 heavy chain interface, were modeled using the MODELLER^18^ web service integrated into UCSF chimera 1.13.1,^19,20^ picking the conformation with the lowest Z-DOPE energy (discrete optimized protein energy) score. The amino acid labeling is following the nomenclature of Zhou et al.^16^ The CDR-H3 of b12 contains a 10 residue insertion, which in the Kabat and Wu numbering are designated as 100a, 100b, ..., and 100j, respectively.^21^

The b12–gp120 protein complex was placed in a triclinic box of dimensions 115.3 Å × 120.2 Å × 125.7 Å; the system was neutralized by adding 14 Cl^–^ ions and solvated with 46 056 TIP3P water molecules.

### Classical Molecular Dynamics Protocol

The AMBER 18 simulation package^22,23^ was chosen to perform all of the classical simulations. To set up the simulation input files, the AmberTools Leap program was used. The simulation parameters used for protein components of the system were obtained from the ff14SB Amber force field,^24^ whereas glycan parameters were taken from the GLYCAM06 force field.^25^ Water molecules were modeled using the TIP3P force field^26^ and the solvated free ions were described by Merz and co-workers.^27^

The first step in the classical set of simulations was a minimization of 2000 cycles, followed by heating gradually from 0.1 up to 298.0 K using 25 000 time steps of 2 fs with NVT ensemble conditions. Next, an NPT ensemble along 0.5 ns (2 fs of time step) at 1 atm and 298 K was applied to reach constant system density. Covalent bonds involving hydrogen atoms were constrained using the SHAKE algorithm,^28^ and long-range electrostatic interactions were treated with particle-mesh Ewald using a real-space cutoff of 10 Å.^29^ The Langevin dynamics was used to heat the system and a Berendsen barostat was used to equilibrate the pressure. Accordingly, a collision frequency of 2 ps^–1^ and pressure relaxation time of 1 ps were applied for the thermostat and the barostat, respectively. An additional equilibration step using the MD approach with an NVT ensemble at 298 K during 0.5 ns (2 fs of time step) was performed. Finally, the system was submitted to a production run of 80 ns with an NVT ensemble at 298 K.

### Accelerated Molecular Dynamics Protocol

The main goal of a long accelerated molecular dynamics^30^ (aMD) trajectory production in this work was to enhance the system sampling to obtain the highest number of possible conformations of the antigen–antibody complex by artificially truncating the energy barriers that separate the different low-energy system states. This technique allows for faster and more extensive explorations than conventional molecular dynamics (cMD); with the correct choice of parameters, it also retains the normal physical properties of the system. The modification of the potential energy in eq 1 consists of truncating the potential when it is below defined threshold energy by adding the new term for the boost potential Δ*V*(*r*)
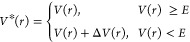
1The boost potential is responsible for modifying the potential energy profile being calculated; it is defined in eq 2.
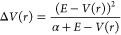
2where *V*(*r*) is the original potential, *E* is the reference energy, and α is the acceleration factor.

All aMD productions were performed over 150 ns using an NVT ensemble and starting from three different snapshots, equally spaced along the last 30 ns of conventional MD (cMD) production; this resulted in a total trajectory of 450 ns. All aMD productions were carried out with an Amber simulation package and the values of the (*E*, α; in kcal/mol) parameter couple used were 12 784.52, 618.4 and −415 123.05, 45 023.7 for the dihedral and system potential, respectively. The parameters’ values were derived following the methodology proposed by Miao et al.^31^ Similarly to the previous cMD simulations, Langevin dynamics^32^ and its thermostat with a relaxation time of 1 ps^–1^ under an NVT ensemble were used.

Clustering of aMD trajectories was performed using the DBSCAN^33^ clustering algorithm as it is implemented in the cpptraj program of AMBER 18^23^ to ensure the aMD convergence. The minimum number of points to define a cluster was set to 9 and the epsilon parameter was set to 1.6. Moreover, the distance metric to be used in the clustering analysis was the root-mean-square deviation (RMSD) value of the whole antigen plus the three CDRs of the antibody. To ensure that clusters found would be consistent across all simulations, a combined trajectory from each one of the three production runs of 150 ns was used and partitioned in strips of 25 ns for its clustering analysis.

### Two-Dimensional Free-Energy Profiles of Protein–Protein Complexes

Two-dimension potential of mean force (2D-PMF) was obtained from reweighting aMD simulations to recover the original free energy profiles of different conformational complexes between Fab domain of an IgG1-b12 antibody and gp120 of the HIV spike protein (hereafter, PMF). Energetic reweighting was conducted using a cumulant expansion to the second-order, which is able to recover the most accurate free-energy profiles within statistical errors.^34^ 2D-PMF profiles were built considering two different variables: (i) the binding free energy (BFE) between the Fab domain of the IgG1 antibody and the gp120 protein of HIV.
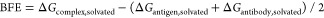
3The BFE between the antigen and antibody was calculated using the molecular mechanics/Poisson Boltzmann surface aea (MM/PBSA) calculations,^35^ as implemented within the AMBER 18 package.^23^ The overall objective of the MMPBSA methodology is to calculate the energy difference between two states, which most often represent the bound and unbound state of two solvated molecules. Energy differences are calculated by combining the gas-phase energy contributions that are independent of the solvent with the solvation energy components (polar and non-polar contributions) derived from an implicit solvent model. This methodology will allow decomposing the energy contribution to the BFE either by residues or by pairs of residues.

(ii) The interface-buried surface (BS) between the antibody and the RBD of gp120 protein was derived by considering the area masked by the solvation effect between the two proteins.
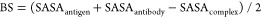
4where SASA refers to the solvent-accessible surface area. SASA values were obtained using the Connolly algorithm^36^ as implemented in the cpptraj tool of the Amber 18 package.^23^

Finally, two different 2D-PMF profiles for all of the protein complexes were obtained, i.e., PMF = *f*(BFE, RMSD) and PMF = *f*(BFE, BS). Calculations were conducted using a bin size of 0.1 Å, 12 Å^2^, and 1.2 kcal/mol for the RMSD, BS, and BFE variables, respectively. The PMF profiles show the dependence between the free-energy landscape of the whole system and some structural system variables. PMF minima show the most stable system conformations when plotted as a function of some structural variables. Considering the focus of this work in the antibody–antigen interface, the RMSD, BFE, and BS variables were used as the best representative of protein–protein interface interactions to build the final PMF profiles.

### Quantum Mechanics/Molecular Mechanics–Molecular Dynamics (QM/MM MD) Protocol

**Figure 2:**
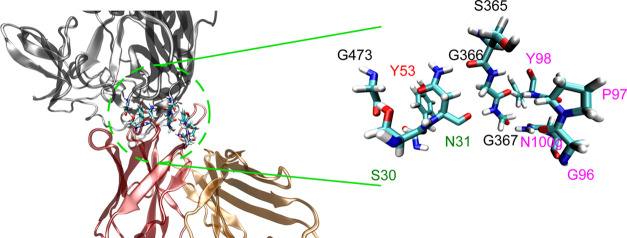
Detail of the QM region of the b12–gp120 interface in the QM/MM MD simulation. The zoomed image presents the three CDR-H chain regions of the b12 protein that bind with the gp120 protein (highlighted by means of a licorice representation). Amino acid labels from the H1 region of b12 are shown in green, H2 in red, H3 in pink, and amino acids from gp120 in black.

Overall, 12 snapshots from the global minimum and adjacent areas (within a PMF radius of 0.015 kcal/mol) of the 1D-PMF profile against the RMSD variable derived from all aMD trajectories were selected. These conformations were used as starting points for ab initio QM/MM MD simulations to relax the interface between the Fab domain of an IgG1 antibody and the RBD of the spike protein of HIV-1 virus. The quantum region was mainly defined by means of a contacting residue analysis on the protein–protein interface and considering crystallographic structural data from previous studies, where the three heavy-chain complementarity-determining regions (CDRs) appear to show a higher degree of interaction with the antigen.^16^ The final stage considered a quantum region containing 158 atoms and was made of 11 amino acid residues, as shown in Figure 2.

All QM/MM MD simulations were conducted by means of the AMBER-PUPIL-NWChem tandem, where the PUPIL program^37,38^ links the classical engine (AMBER 18) with the quantum engine (NWChem^39^) by calculating and managing all of the QM/MM coupling within the QM/MM MD approach. All starting conformations were subjected to the same protocol along 0.6 ps of equilibration followed by 5 ps of production run under NVT dynamics. The usable data were collected from a total production run of 60 ps of the QM/MM MD trajectory. An NVT ensemble at 298 K with a temperature regulation using a Langevin thermostat (collision frequency of 10 ps^–1^) and a time step of 1 fs was used. The remaining simulation parameters and force fields used for the QM/MM MD simulations were the same as those used for a classical MD protocol. The MM region was parameterized using the ffSB14 Amber force field,^24^ while the QM region was modeled by means of density functional theory (DFT) using the M06-2X functional^40^ in combination with a 6-31G basis set for all quantum atoms. It should be noticed that medium-range (i.e., ≤5 Å) noncovalent interactions, such as conventional (e.g., N–H···O and H–O···H) and nonconventional (e.g., C–H···O) hydrogen bonds, are better described by the M06-2X functional than usual DFT functionals.^41^ Long-range electrostatic interactions in the QM regions were considered by means of Ewald summations with a real-space cutoff of 21 Å within the QM/MM coupling methodology.

## Results

### RMSD/Root Mean Square Fluctuation (RMSF) Analysis of the Protein–Protein Complex

Figure S1 shows the root mean square distance (RMSD) along the 80 ns of cMD of the complex system made of the IgG1-b12 antibody linked with the gp120 protein of the HIV spike glycoprotein. More specifically, the variable domain on the Fab region of the IgG1-b12 antibody (b12–gp120 protein complex) was considered. The simulated system reached a plateau after 20 ns of production MD. The average RMSD in the last 60 ns of production was 3.15 ± 0.26 Å; the low standard deviation shows a stabilized system along the time period of this production run. However, relative movement of the residues involved in the protein–protein interface are important since it facilitates the formation of asymmetric interfaces among different protein domains.^42^ The flexibility of the b12–gp120 interface was studied by means of the root mean square fluctuation (RMSF), which measures the amplitude of atom motions during cMD simulation and thus, the residue specific flexibility. Figure S2 shows the RMSF obtained considering all atom fluctuations of each residue of the b12–gp120 complex along 80 ns of cMD, starting from crystallographic coordinates. The HIV-gp120 protein does not show large fluctuations compared with those observed in the Fab domain of the IgG1-b12 antibody. Indeed, the gp120 protein region has the highest residue fluctuation involving the amino acids around L125 (RMSF ≈ 11.2 Å), whereas a different behavior is observed for the Fab domain of the IgG1-b12 antibody. Interestingly, the antibody Hc chain section involving the three CDR-H regions is much less flexible (2.7 Å < RMSF < 10.6 Å) when compared to the last half of the chain, starting from G106 (Hv) amino acid (11.0 Å < RMSF < 16 Å) and the whole Lc chain. The latter constitutes the most flexible region of the system (9.0 Å < RMSF < 17.6 Å); this region contains the three CDR-L regions.

### Conformational Scanning of the Protein–Protein Complex

An accelerated molecular dynamics (aMD) approach was used to scan the conformational landscape of the protein complex between the HIV-g120 spike protein and the IgG1-b12 mAb.^30^ The main advantage of this technique is a quicker and more extended biological sampling of the conformational space that is difficult to replicate with standard classical molecular dynamics (cMD). It is known that the time scale required to address such large systems using the cMD approach can be shortened to just hundreds-of-nanoseconds using an aMD approach.^43,44^ aMD convergence was ensured by means of a clustering analysis along the combined production runs of all three aMD simulations.^45^Figure S3 shows how the total number of clusters considering strips of 25 ns of trajectory was kept around five to six clusters along the last 75 ns of trajectory.

Figure S4 shows the three PMF profiles plotted against the radius of gyration of three proteins, i.e., the antigen, the antibody, and the three CDRs located in the heavy chain of the antibody. PMF figures only show the region with Δ*G* ≤ 5.0 kcal/mol for the sake of clarity. It is observed that when doing a more exhaustive sampling of the systems using the aMD methodology, the shapes of the three systems do not vary much along all of the conformational study (*R*_g_ = *R*_g,min_ ± 0.7 Å, where *R*_g,min_ refers to the *R*_g_ value at the global minimum). In fact, the *R*_g,min_ values obtained by aMD for the three studied PMF profiles are located near the averaged values derived from the cMD trajectories, (vertical dashed line) which were derived from a much less exhaustive exploration of the conformational space. In all three cases, the distance between the *R*_g,min_ and the averaged value of MD (*R*_g,MD_) is less than 0.5 Å, which indicates a small structural variation between the averages of cMD and aMD. However, this difference is much smaller when only the three CDRs of the antigen heavy chain are considered (where the greater weight of the antibody–antigen interaction is located). This indicates that there is hardly any structural distortion in the antigen recognition region, and therefore no significant loss of functionality is expected throughout all simulations.

**Figure 3:**
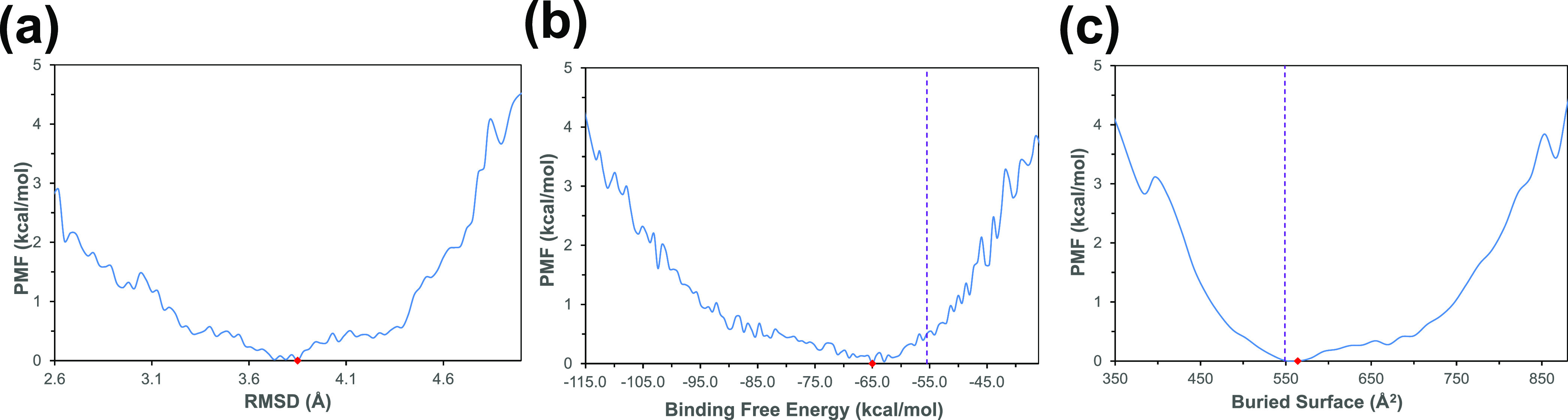
Potential of mean force (PMF) plots showing the dependence between the free-energy landscape and the (a) root mean square displacement (RMSD), (b) binding free energy (BFE), and (c) buried interface surface of b12–gp120 protein–protein complex (BS). For each PMF, the absolute minimum is also shown (red point). The vertical dashed line represents the averaged value of the *x*-axis variable along the previous cMD simulation. The RMSD value from the cMD simulation is not shown (out of scale at 2.24 Å).

On the other hand, the PMF profile along the binding free energy (BFE) of the protein complex (Figure 3b) provides information about the interaction energy of the complex for the more populated conformations. In this case, there is a greater variation between the global minimum of the PMF profile with respect to the average value derived from the cMD simulations; this corroborates that the aMD approach samples with less computational effort than the cMD for the regions of minimum energy. Regarding the PMF profile along the RMSD variable (Figure 3a), the average of cMD (RMSD_cMD_ = 2.24 Å) is far from the PMF global minimum (RMSD_aMD_ = 3.85 Å). This makes the RMSD variable useful for sweeping and better discerning between the different conformations around the global minimum of the PMF profile of the complex. Despite the conformational variability when the complex is represented as a function of the RMSD, the global minimum considering the buried surface (BS) between the antigen and the antibody (Figure 3c) is very close to the average value obtained by cMD once the trajectory is stabilized (dashed blue line in Figure 3c).

**Figure 4:**
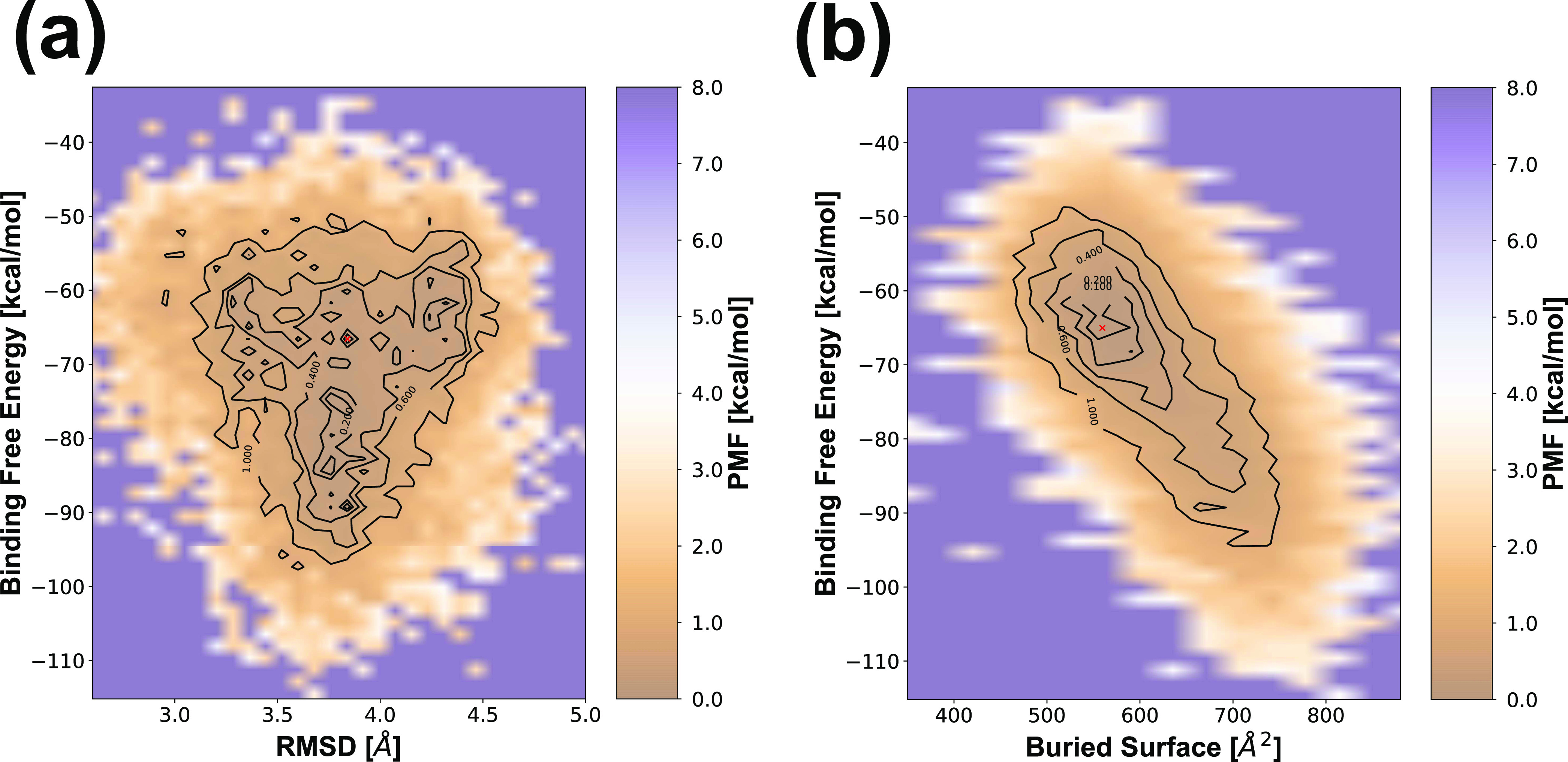
Plot of the two-dimensional potential of mean force (2D-PMF) is showing the landscape between the binding free energy (BFE) and the (a) root mean square displacement (RMSD) and (b) buried interface surface (BS) of the b12–gp120 protein–protein complex. Absolute minima are marked with a red cross.

Furthermore, analyses of the 2D-PMF profiles correlated by the pairs of the three studied variables, i.e., BFE, RMSD, and BS, were performed. Figure 4 shows the two-dimensional profiles PMF = *f*(BFE, RMSD) and PMF = *f*(BFE, BS) of the b12–gp120 protein–protein complex. The free-energy profile of the energy reweighting was recovered along the whole 450 ns of aMD simulations (see the Methods section).

The 2D-PMF profile as a function of the BFE-RMSD variables is plotted in Figure 4a. The absolute minimum is located at the point −66.6 kcal/mol; 3.84 Å, which is quite close to the RMSD and BFE values of the absolute minima of the 1D-PMF profiles reported above (Figure 3a), i.e., BFE = −65.0 kcal/mol and RMSD = 3.85 Å. It should be noted that this profile presents an important range of possible BFE values (from −65 kcal/mol up to a more favorable value of −90 kcal/mol) for very similar values of RMSD ∼ 3.84 Å. However, decreasing BFE results in less structural variability of the system (lower dispersion of expected RMSD values).

In addition to the previous profile, the 2D-PMF profile as a function of the variables (BFE; BS) is shown in Figure 4b. In this case, the absolute minimum is located at the point −65.0 kcal/mol; 559.5 Å^2^. These coordinate values are still much closer to their corresponding minima of the 1D-PMF profiles, i.e., BFE = −65.0 kcal/mol and BS = 563.9 Å^2^. This behavior is expected given the high correlation between the BFE and BS variables in terms of the antibody–antigen interaction. It is observed that the stabilization of the interface with a more negative BFE is mainly due to an increase in the interaction surface of the complex (higher BS). However, the most likely conformation of the profile (BFE; BS) is still around −65 kcal/mol.

### Quantum Relaxation of the b12–gp120 Interface

**Figure 5:**
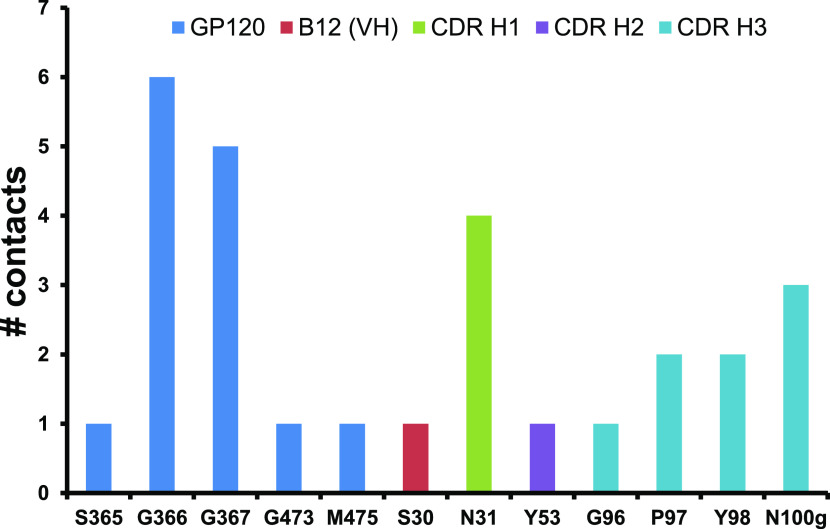
Number of contacts by residue along the protein–protein interface of the b12–gp120 system. A residue contact is defined as a distance of less or equal than 4 Å between two atoms of residues located on both sides of the interface with a population greater than 90% among all conformations around the global minimum of the PMF = *f*(RMSD) profile (free energy lower than 0.1 kcal/mol), which were derived from aMD trajectories. Residues belonging to the CDR regions of both mAbs are shown.

To conduct QM/MM MD calculations to study the IgG1–gp120 interface, the amino acids that are likely to play the most important role in the antibody–antigen interface interactions were determined. Figure 5 shows the contacts at the b12–gp120 interface. Two residues at both sides of the interface were considered to make a contact when two atoms of their main chain are kept at a distance of less or equal than 4 Å between them in more than 90% of all conformations around the global minimum of the PMF = *f*(RMSD) profile. This profile presents a set of conformations with a wide range of binding energies around its global minimum, as seen above (Figure 4a). The most representative residues making contacts in the interface along all of these conformations might play a significant role in the potential interactions at room temperature of the interface between the IgG1 and gp120 proteins. Thus, these residues should be considered using a QM level of theory in the QM/MM MD simulation.

Figure 5 shows the most representative amino acids making close contact with other amino acid residues between both sides of the interface. Five residues from the gp120 antigen’s protein, i.e., three residues from the “CD4-binding loop” region (i.e., S365, G366, G367) and two from the “outer domain-exit loop” (i.e., G473 and M475), were determined as representatives on the interface contacts. In this work, the secondary structural elements for the b12–gp120 complex conformation are set following a previous convention published by Kwong et al.^10^ The CD4-binding loop region contains the secondary structural elements β15 and α3, whereas the outer domain-exit loop is defined as the region between β24 and α5 (Figure S5). Residues G366 and G367 from the outer domain-exit loop of the gp120 protein have the highest number of persistent contacts along the most populated conformations. On the other hand, the amino acids from the IgG1-b12 antibody that are more involved in the contacts with the antigen are mainly localized in the CDR-H3 region (G96, P97, Y98, and N100g) (Figure S6) and, to a lesser extent in the CDR-H1 and H2 with only one amino acid each one, the N31(H1) and Y53 (H2), respectively. Similar to what was observed experimentally in the crystal structure,^16^ no representative contacts were obtained in the CDR domain of the IgG1 light chain. The Kabat numbering scheme to label the amino acids belonging to the CDRs of mAb IgG1-b12 is used, and these were obtained from the SabDab structural antibody database.^46,47^ The highest numbers of contacts involving the IgG1-b12 antibody are localized on the polar amino acids N31 (H1) and N100g (H3). Thus, persistent hydrogen bond formation across the interface involving these residues is expected.

The 12 snapshots extracted from the global minimum and the two adjacent local minima of the PMF profile plotted against the RMSD variable (Figure 3a) were used as starting points for ab initio QM/MM MD simulations. These conformations sample a short range of RMSD values (∼3.72: 3.85 Å) but a relatively wider range of BFE (∼57.5: 87.5 kcal/mol), as shown in Figure S7. The closest persistent amino acids along the interface obtained from previous contact analysis, and its neighbor residues, were made quantum (QM region) (Figure 2). The influence of the environment surrounding the QM region was considered by modeling the rest of the system using a classical molecular mechanics approach (MM region). The M06-2X density functional, by explicitly simulating electrons, can better account for not only electrostatic interactions but also van der Waals interactions and short-range dispersion forces that are not considered in classical force-field simulations where atoms have fixed partial charges.^41^ Following the protocol defined in the Methods section, each one of the system starting point underwent 5 ps of hybrid MD to relax the interface between the Fab domain of the IgG1 antibody and the RBD of the spike protein of HIV virus. Joining all trajectories, a total amount of 67.2 ps of the QM/MM MD trajectory was obtained and used for the analysis and comparison of the main interaction with the classical trajectories.

### Main Interactions on the b12–gp120 Interface

The most persistent and populated polar interactions involving the hydrogen bonding (HB) network and salt bridges (SB) under physiological conditions at room temperature were studied. Table 1 summarizes the major interactions between the gp120 spike protein of the HIV virus and the mAbs IgG1-b12. The comparison includes five sets of different conformations: *I*_X-ray_, the interactions collected from a crystallographic structure; *I*_MD_ the interactions obtained from the conformation stored along the cMD trajectory; *I*_RMSD_ the main interactions of the conformational set around the absolute minimum of the 2D-PMF profile, considering the correlation between the binding free energy (BFE) of the complex with the RMSD variable; *I*_BS_ as the conformational set around the absolute minimum of the 2D-PMF profile correlating BFE and BS variables; and finally, *I*_QMMM_, the main interactions obtained from the QM/MM MD trajectories relaxing a conformational set around the minimum of 2D-PMF profile (BFE,RMSD). In all cases, only those interactions that were conserved throughout the conformational set with a population of 33.3% or higher have been considered.

**Table 1:** Hydrogen Bonds and Salt Bridges of the b12–gp120 Interface^a^

IgG1-B12		GP120							
Res	Atm	Res	Atm	type	*I*_X-ray_^b^	*I*_MD_	*I*_RMSD_	*I*_BS_	*I*_QMMM_
*heavy chain*									
R28	NH1	N280	O	HB	2.39				
S30	OG	G473	O	HB		2.70(0.12)	2.73(0.11)	2.71(0.12)	2.71(0.13)
N31	ND2	S365	O	HB	2.80	2.86(0.08)	2.85(0.09)	2.86(0.08)	2.83(0.10)
N31	O	G367	N	HB	2.85	2.84(0.08)	2.85(0.09)	2.84(0.08)	2.80(0.10)
Y53	O	M475	N	HB		2.89(0.08)	2.87(0.08)	2.88(0.07)	2.87(0.08)
Y98	N	G366	O	HB	2.80	2.86(0.08)	2.86(0.08)	2.86(0.08)	2.80(0.10)
Y98	OH	R419	NH1	HB		3.51(0.29)	3.38(0.31)	3.41(0.33)	3.41(0.37)
Y98	OH	R419	NH2	HB	3.67		3.29(0.34)	3.38(0.33)	3.39(0.42)
W100	O	R419	NH1	HB	2.36	2.83(0.09)			
W100	O	R419	NH2	HB		2.84(0.09)			
N100g	ND2	G367	O	HB	2.30	2.81(0.09)	2.82(0.09)	2.82(0.09)	2.84(0.09)
Y100h	OH	D368	OD1	HB	2.39	2.71(0.13)	2.73(0.12)	2.72(0.13)	
Y100h	OH	D368	OD2	HB		2.70(0.13)	2.73(0.13)	2.72(0.13)	2.59(0.11)
E58	OE1	K432	NZ	SB		3.08(0.38)			3.11(0.38)
E58	OE2	K432	NZ	SB		3.06(0.36)			2.97(0.26)

a Data derived using AMBER software. Standard error is shown in parentheses.

b Crystallographic data from Zhou et al.^16^ and processed with the AMBER package.

The crystallographic structure (*I*_X-ray_) contains eight different HBs at the interface of the b12–gp120 complex. These interactions are mainly found between the CDR-H3 of b12 and the CD4-binding loop of gp120 (strand β15), strand β19, and loop LD. Interestingly, two of the crystal HBs, i.e., R28(Hv)···N280 and W100(H3)···R419, are lost due to the low persistence of these interactions under physiological conditions in the conformational scan around the global minimum of both 2D-PMF profiles and *I*_QMMM_. In most of the observed conformations, the CDR-H3 tip is projected toward the glycosylated face, with W100(H3) of the CDR-H3 tip sandwiched between the two strands β17 and β19 of gp120 (i.e., R419 and N386 residues), as reported from crystallographic data.^16^ However, the HB interaction W100(H3)···NAG described by X-ray data is lost in most of the more populated conformations in solution.

**Figure 6:**
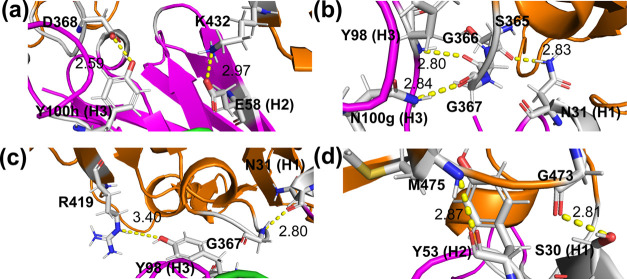
Detail of the interface polar interactions between the spike gp120 protein of HIV virus and the mAb IgG1-b12, derived from QM/MM MD trajectories. (a) HB8(Y100h-D368) left and SB1(E58-K432) right. (b) HB5(Y98-G366) top left, HB7(N100g-G367) bottom left, and HB2(N31-S365) right. (c) HB6(Y98-R419) left and HB3(N31-G367) right. (d) HB4(Y53-M475) left and HB1(S30-G473) right. Residues involved in HB and SB interactions are shown as sticks at the interfaces. Amino acids from three CDRs on the heavy chain of b12 that are involved in interface binding are indicated by H labels in parenthesis. HIV-gp120 is colored orange and the heavy chain of IgG1-b12 is pink.

On the other side, two new HBs appear in all of the populated conformational sets involving the CDR-H1 and -H2, i.e., S30(Hv)···G473 and Y53(H2)···M475, with the outer domain-exit loop (Figure 6b). The rest of HBs are preserved along the conformational sets studied, the HB (Y100h (H3)···D368), between the CDR-H3 and the outer domain-exit loop of gp120, the strongest HB observed (average bond distance of 2.59 Å, Figure 6a). After relaxing the system using the QM/MM MD approach, an additional persistent SB is formed between the CDR-H2 and strand β21 (E58(H2)···K432, Figure 6). In fact, polar interactions between CDR-H2 and gp120 are strengthened due to the additional HB and SB formation, whereas CDR-H3 loses one polar interaction when the most populated solvated conformations and crystal proteins are compared.

**Figure 7:**
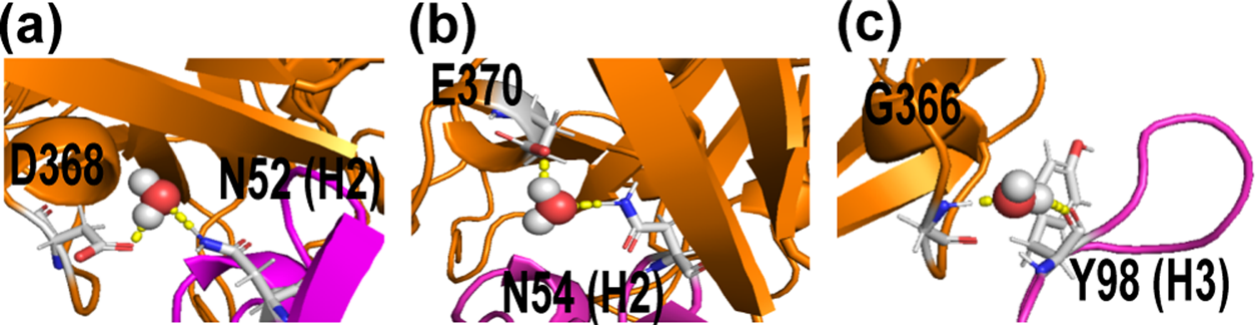
Representative images of the relevant water-bridged complexes of the b12–gp120 interface: detail of the CD4-binding loop residues from gp120 interacting with (a) N52 (H2), (b) N54 (H2), and (c) Y98 (H3) of the b12 protein. Data derived from the *I*_QMMM_ conformational set.

In addition to polar interactions, Table S1 lists the most persistent water-bridged complexes between residues at both sides of the interface. Thus, starting from the initial structure of the crystal, classical MD simulation (*I*_MD_) gives rise to four water-bridged complexes between the CDR-H2 and -H3 of b12 with the strands β15, loop LB, and with the outer domain-exit loop (Table S1) of the gp120 protein. In the most populated conformations of *I*_RMSD_ and *I*_BS_, these water-bridged complexes decrease and are less persistent throughout the studied conformations. However, the relaxed conformations at a QM/MM MD level (*I*_QMMM_) show that these water-bridged interactions are recovered (Figure 7). The introduction of these additional interactions will provide additional stability to the interface between IgG1-CDR and the CD4-binding loop of the gp120 protein.

### Binding-Free-Energy Decomposition

**Figure 8:**
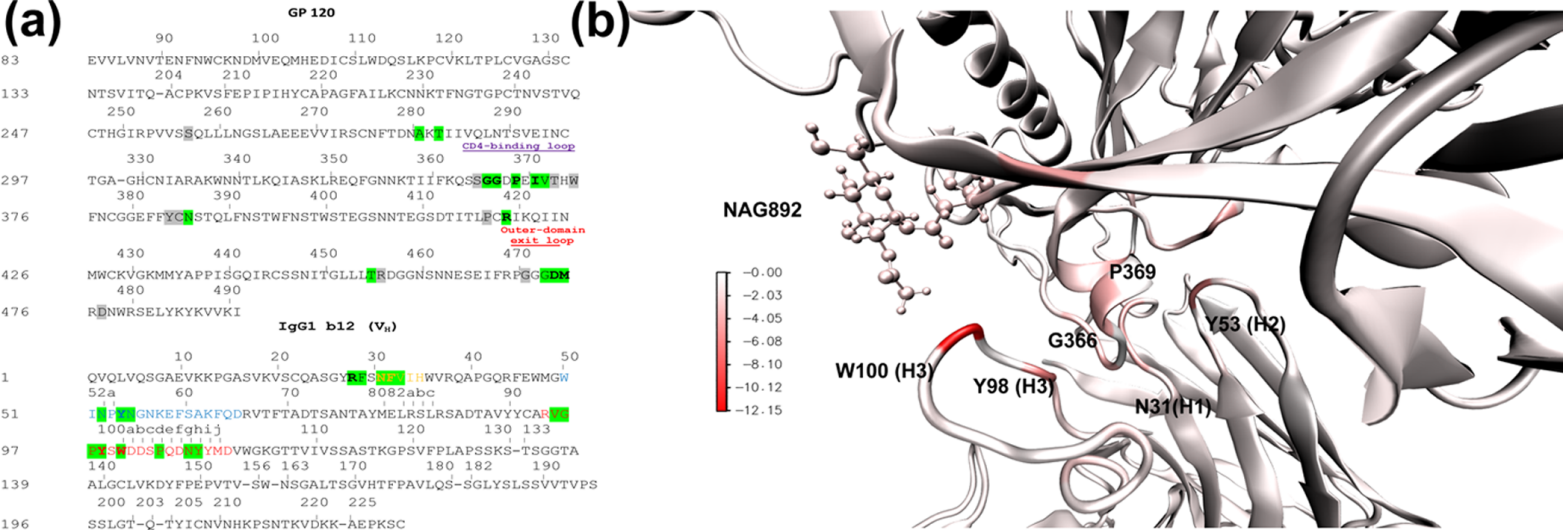
Comparison of (a) main interacting residues on the epitope of gp120 and the paratope of the b12 heavy chain. The color range for residue contribution to the BFE: <−2.0 kcal/mol (in bold and highlighted in green), contribution range −1.0: −2.0 kcal/mol (highlighted in green), and contribution range −0.25: −1.0 kcal/mol (highlighted in gray). (b) Surface energy contribution to BFE per residue of RBD complexed with IgG1-b12. Energy contribution is denoted by a color map.

The BFE decomposition per residue and by pairwise residue allows us to better understand the contributions of the main residues to the stability of the b12–gp120 complex, that is, the role played by each residue involved in the different polar (e.g., SB and HB) and hydrophobic interactions. Tables S2 and S3 list all of the independent residue contributions to the overall BFE, while Table S4 displays the pairwise residue contributions to the BFE of those pairs located at both sides of the interface. Energy decomposition analyses were conducted on the conformational sets around the global minimum of both 2D-PMF profiles (i.e., *I*_RMSD_ and *I*_BS_). Figure 8 shows the most persistent paratope and epitope residues throughout the present conformational study, as well as a color surface map on the residues’ contribution to the BFE of the complex. Figures S5 and S6 show the predominant secondary structure along the b12–gp120 complex sequence on the *I*_MD_ and *I*_QMMM_ sets, which is compared with the experimental reported secondary structure of the crystal.^10,16^ No significant secondary structural differences among the three compared structures are observed.

W100 (H3) is the amino acid with by far the greatest contribution to the BFE (−11.7 and −12.2 kcal/mol for the *I*_RMSD_ and *I*_BS_ conformational sets, respectively, Table S2). This high level of stabilizing contribution is explained by the existing interactions of this amino acid with the β19 strand (R419), and to a lesser extent with the β17 strand (N386), as well as with the anchored glycan to N386 (NAG892) (Table S4). The crystal structure presents the same interactions involving W100(H3) and is preserved along all of the most populated conformations, thus becoming one of the most important points of the b12–gp120 antibody–antigen interface.^16^ In fact, a comparison between all residue contributions of different b12–CDRs and the BFE (Table 2) reveals a contribution ratio of ∼10:10:30 for the H1/H2/H3 relationship in the *I*_RMSD_ conformational set, while the conformational set *I*_BS_ presents a ratio of ∼10:7:31. The latter is quite similar to that previously reported from alanine scanning calculations with a final ratio of approximately ∼10:7:21, for the same concept.^48^ Similarly, Zwick et al.^49^ showed the importance of this domain compared to the other two, i.e., H1 and H2 domains, and the important role played by Y53 (H2), Y98 (H3), and W100 (H3) residues in the b12–gp120 complex’s stability. Indeed, these amino acids are those that present a higher contribution to the BFE of the b12–gp120 complex (Table S2). After binding-free-energy decomposition on a pairwise per-residue basis (Table S4), the interactions’ breakdown can be observed in more detail. Focusing on the previously described residues, W100 (H3) is the one that presents the highest energetic interactions, mainly with R419, N386, and NAG892. The Y98 (H3) residue presents less important interactions than W100 (H3), but involves some residues from the CD4-binding loop (i.e., G366, P369, and D368). Finally, residue Y53 (H2) interacts mainly with the outer domain-exit loop (mainly D474 and M475 residues) and to a lesser extent with the CD4-binding loop (I371).

**Table 2:** Details of the Energy Contribution to the Binding Free Energy of Residues Belonging to Some Regions of the b12–gp120 Complex (in kcal/mol)

		*I*_RMSD_	*I*_BS_
b12	pre-H1^a^	–3.4	–2.2
	H1	–7.6	–7.3
	H2	–7.8	–5.3
	H3	–22.3	–22.8
gp120	LD	–2.0	–1.9
	CD4-binding loop	–14.4	–14.1
	β17−α4	–3.0	–3.0
	β19	–3.8	–3.7
	β20−β21 loop	–0.6	1.1
	β23	–2.1	–1.8
	outer domain-exit loop	–9.3	–8.6

a Four residues previous to the CDR-H1 domain (i.e., Y27, R28, F29, and S30).

The main regions of the interaction of gp120 are more varied than those of IgG1. The contributions to the overall BFE that each secondary structural element presents (once the individual contributions of all amino acids are added, which they are composed of, Table 2) show the relative importance for each one of them. Thus, the ratio of contributions is 2:2:3:4:9:15 between the different contact regions LD/β23/β17−α4/β19/outer domain-exit loop/CD4-binding loop, arranged in order of importance.

## Discussion

The interface between the IgG1-b12 antibody and the gp120 protein of the HIV-1 spike protein has been examined using an aMD approach, which allows scanning a wide range of conformations under physiological conditions at room temperature. After energy reweighting of aMD trajectories to obtain 1D- and 2D-PMF profiles, it has been possible to discern the more populated conformations located at the global minimum of the original free-energy profiles of the b12–gp120 complex. Two two-dimensional profiles have highlighted (i) the relationship between the BFE of the b12–gp120 complex and the RMSD variable of the system and (ii) the relationship between the BFE and the BS of the studied antibody–antigen interface. Both 2D-PMF profiles are well correlated, making it easy to identify the set of conformations around the global minimum of both profiles. On the one hand, the *I*_RMSD_ set represents the most populated conformations that support a very similar BFE, as well as a similar geometric distance to the crystal structure. On the other hand, the *I*_BS_ set shows those conformations of the most populated antibody–antigen complex with similar BFE and contact surfaces.

Contact analysis allows the identification of those amino acids that could play an important role in the interactions along the interface. That is, those that exhibit closer proximity as well as contact persistence along the most populated conformations in the aMD trajectories. Some residues from the CDR-H3, and to a lesser extent, from the CDR-H2 and -H1 domains of b12 present persistent contacts with residues from the CD4-binding loop and the outer domain-exit loop of gp120 (Figure 5). These amino acids deserve to be modeled at a higher chemical level; in this way, the possible polar and hydrophobic interactions throughout the simulation can be better represented. The conformations around the global minimum of 1D-PMF with respect to the RMSD variable were allowed to relax using a QM/MM MD method (considering the residues with a higher number of persistent contacts along the interface as belonging to the quantum region). This relaxation leads to a reinforcement of the interactions between gp120 with CDR-H2 in detriment to those existing with CDR-H3 observed in the crystal structure.

IgG1-b12 binds preferentially to the outer domain of the gp120 protein.^16^ The literature mainly shows two binding sites of the b12 antibody to the gp120 protein, i.e., the CD4-binding loop (β15−α3 region, spanning residues 364–373) and the outer domain-exit loop (β24−α5 region, spanning residues 470–476). These regions are shown as the most important for the stabilization of the b12–gp120 complex, not only in the crystal structure^16^ but also in the simulations described in this work under physiological conditions at room temperature (Table 2). However, other observed epitope regions of gp120, i.e., strand β19 and β17−α4, even though they are less important from an energetic point of view, play an important role in stabilizing the binding with the tip of CDR-H3 (W100).

We investigated the b12–gp120 interface regarding the most persistent polar interactions (Table 1). It is observed that under physiological conditions, the interaction between the gp120 protein and the CDR-H1 and -H2 is enhanced, as opposed to the polar interactions with CDR-H3 (Table 1). More specifically, the distribution of persistent polar interactions is inverted, going from an initial 3:5 ratio of polar interactions between the CDRs (H1 + H2) and H3 of the crystal to a final value of 5:4 in the *I*_QMMM_ set. However, a more equitable distribution of contributions to BFE between CDRs (H1 + H2) and H3 is obtained when all of the interactions are considered instead of the most persistent polar interactions only (Table 2). These results are similar to the ones reported by Burkovitz et al.^48^ by means of an alanine scanning study of the crystal structure. The authors reported a ∼10:7:21 relationship on the H1/H2/H3 contribution to the BFE; meanwhile, in the current work, we observed that a relationship of ∼10:10:30 and ∼10:7:31 was obtained for the *I*_RMSD_ and *I*_BS_ sets, respectively, under the simulated physiological conditions of the described simulations. Indeed, a small reinforcement of the CDR-H3 contribution compared to the reported crystal structure contributions was observed.^48^

BFE decomposition on a per-residue basis and on a pairwise per-residue basis was obtained (Tables S2–S4). On close inspection, the paratope of b12 and the epitope of gp120 were determined; as shown in Figures 8 and S6, the regions containing the main residues that are preserved along the studied conformational sets are highlighted. The strongest anchor points of the paratope are found in the amino acids W100 (H3), Y98 (H3), and Y53 (H2). The first one is located at the tip of the CDR-H3 region, anchored between the β19 and β17 regions of the gp120, thus facilitating the interaction of the remaining amino acids of the CDR-H3 with the CD4-binding loop, such as the Y98 (H3). W100 (H3) has lost the HB formed at the crystallographic level with R419 (strand β19) but still remains as the amino acid with the highest contribution to BFE. A second important anchor point appears between gp120 and the b12 residue Y53 (H2); this protrudes from the CDR-H2 pointing toward the outer domain-exit loop, interacting strongly with M475 and to a lesser extent with D474, which likely stabilizes the interface structure around the main anchor point. Similarly, CDR-H1 presents an important binding interaction, close in magnitude to what is observed with the CDR-H2 but slightly more dispersed, involving several residues and focused mainly toward the region of the CD4-binding loop (i.e., N31 (H1) presents the strongest interaction; Table S4). In addition, small but important contribution to the BFE are made by the amino acids (residues 27–30) next to the CDR-H1 that also reinforce its binding, e.g., R28 (Hv), which binds strongly (3.1 kcal/mol) with T455 (strand β23) of gp120.

The close proximity between the CDR-H2 and -H3 of IgG1 with the CD4-binding loop of gp120 leads to a high number of contacts between the different amino acids of both proteins, including those polar interactions mentioned above, as well as important hydrophobic interactions. This proximity gives rise to additional stabilization due to the formation of persistent water-bridged complexes involving amino acids at both sides of the interface (Figures 7 and S8 and Table S1). Interestingly, CDRs of IgG1 mainly form persistent water-bridged complexes with the residues located on the CD4-binding loop of gp120 with populations higher than 25%.

Overall, the studied HIV-1 RBD system bound to the broadly neutralizing antibody IgG1-b12, together with the key binding residues and interactions identified in this work, provide new insights into our understanding of the mechanisms of HIV-1 neutralization and the potential development of better and more precise immunoassays based on the antibody-mediated immobilization. This knowledge could facilitate the development of new treatments^7^ using broadly neutralizing antibodies against HIV-1.^50^

## Supporting Information Available

RMSD and RMSF analyses, cluster convergence plot of aMD trajectories, 1D-PMF profiles along the radius of gyration of different complex parts, 2D-PMF profile as a function of BFE and RMSD variables, relevant water-bridged complexes of the b12–gp120 interface, details of the secondary structure sequence of gp120 and b12, population list of the most persistent water-bridged complexes, and decomposition of the binding free energy on a per-residue and pairwise per-residue basis (PDF)
GP120_B12_pdbs (ZIP)

## Notes

The software packages of AMBER 18 (http://ambermd.org/) and NWChem (https://www.nwchem-sw.org/) were provided by the Barcelona Supercomputing Centre, while UCSF Chimera (https://www.cgl.ucsf.edu/chimera/) and MODELLER (https://salilab.org/modeller/) programs were obtained through a noncommercial license from their webpages. The PUPIL program is openly available for download at http://pupil.sourceforge.net/. The data underlying this study and PDBs of initial cMD and QM/MM-MD simulations structures are available in the published article and its online supplementary material. Additional data that support the findings of this study are available upon request from the authors.
