# Supplementary material for: IgG1-b12–HIV-gp120 Interface in Solution: A Computational Study
Source: J Chem Inf Model. 2021 Dec 31;62(2):359–71. doi: 10.1021/acs.jcim.1c01143 (PMC8790758; doi:10.1021/acs.jcim.1c01143)
Supplement: Supplementary file 1 — ci1c01143_si_001.pdf [file ci1c01143_si_001.pdf]

Supporting Information

The IgG1 b12 − HIV gp120 interface in solution: a computational study

Didac Martí,[1,2] Carlos Alemán,[1,2,3,] Jon Ainsley,[1,4] Oscar Ahumada,[5] Juan Torras[1,2,*]*

[1] Department of Chemical Engineering (EEBE). Universitat Politècnica de Catalunya, C/Eduard Maristany 10-14, Ed I2, 08019, Barcelona, Spain

[2] Barcelona Research Center for Multiscale Science and Engineering, Universitat Politècnica de Catalunya, C/Eduard Maristany 10-14, 08019, Barcelona, Spain

[3] Institute for Bioengineering of Catalonia (IBEC), The Barcelona Institute of Science and Technology, Baldiri Reixac 10-12, 08028 Barcelona, Spain

[4] Evotec Campus Curie, 195 Rte d'Espagne, 31100 Toulouse, Occitanie, France

[5] Mecwins S.L., Parque Científico de Madrid PTM, C/Santiago Grisolía 2, Tres Cantos, Madrid, 28760, Spain

***Corresponding Authors:** carlos.aleman@upc.edu and joan.torras@upc.edu

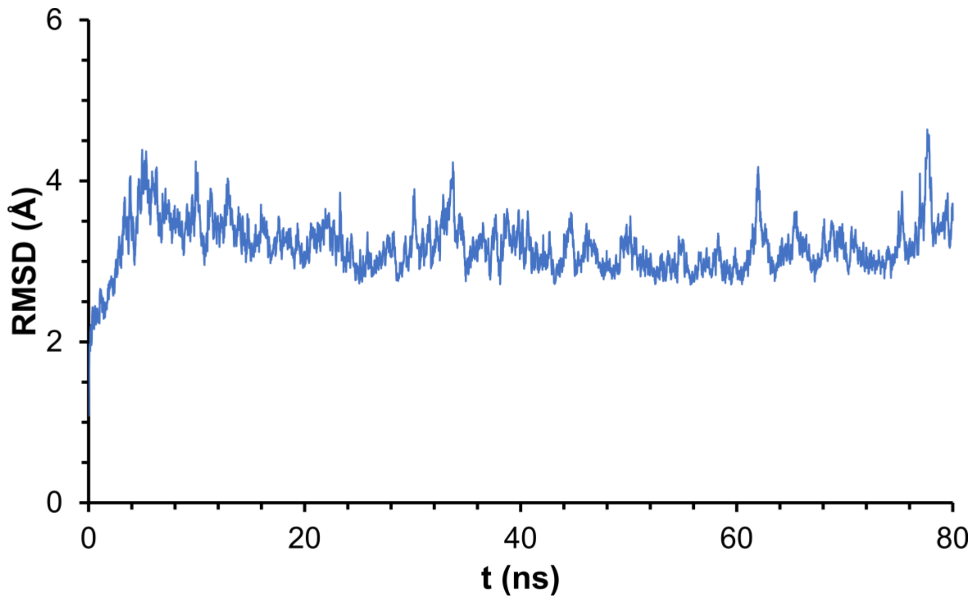

Figure S1. Temporal evolution of the root mean square deviation (RMSD) calculated using all atoms for the cMD simulations of IgG1-gp120 protein complex conducted at 298 K

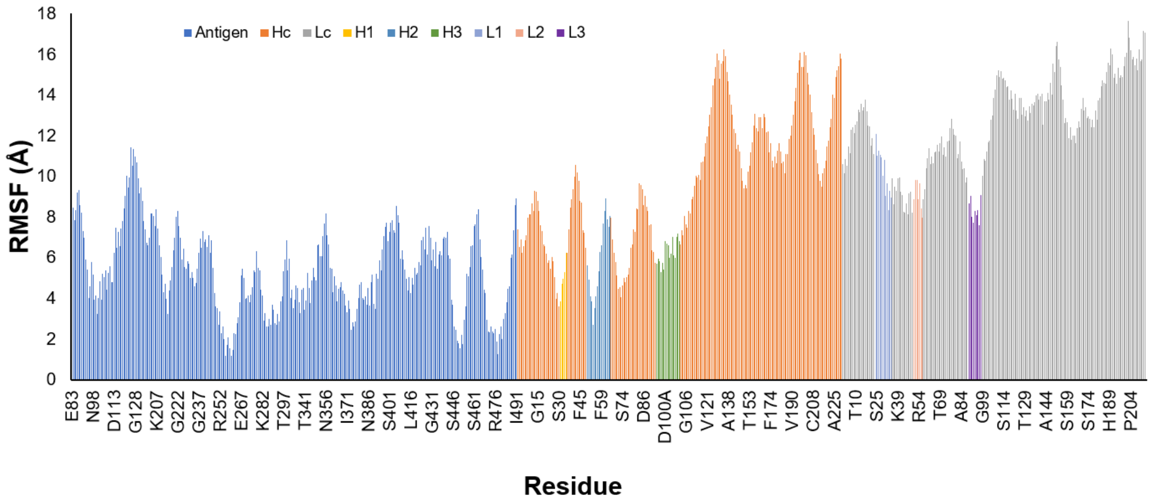

Figure S2. Root mean square fluctuation (RMSF) analyses of the IgG1-gp120 protein complex calculated using all atoms for the molecular dynamics simulations at 298 K. The variable heavy chain (VH) and the variable light chain (VL) from Fab antibody domain, the gp120 protein (HIV), and the six region binding domains (RBDs) from the spike glycoprotein of HIV, are shown.

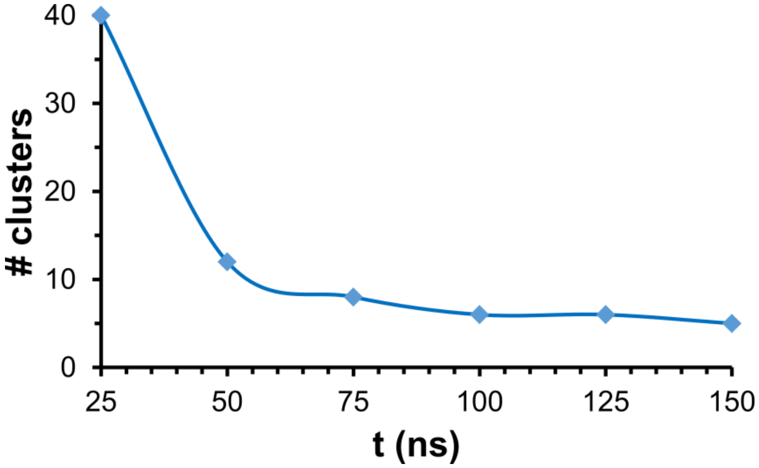

Figure S3. Clustering analysis of all combined aMD trajectories is plot. The total number of clusters obtained along the trajectory in strips of 25 ns, are shown.

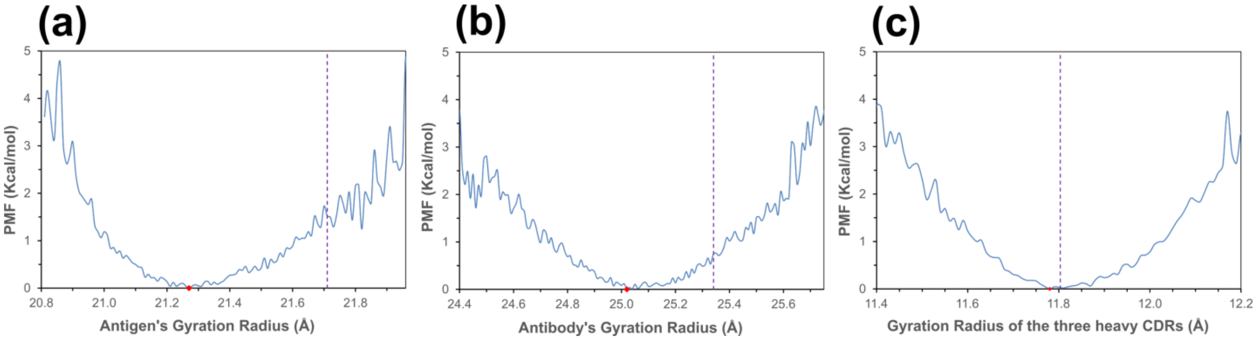

Figure S4. Potential of mean force (PMF) showing the dependence between the free energy landscape and the radius of gyration of the (a) antigen, (b) antibody, and (c) the three heavy CDRs of b12–gp120 protein-protein complex. The absolute minima are also shown (red diamond). Vertical dashed lined represent the averaged value of X-axis variable along the classical MD simulation.

GP 120

```
            β0   β1                    β1a
                 90         100        110       120        130
                 |          |          |         |          |
83          EVVLVNVTENFNWCKNDMVEQMHEDICSLWDQSLKPCVKLTPLCVGAGSC
MD              TT   BBBBTTTS GGGT    TTTTBBB E    SS       SSS T
QMMM         BB   S   BBBBTTTS HHHHH   HHHHB B    B      S    STTTT

                          β3a  β4         β5     ℒA     β6   β7
                 204    210      220        230       240
                  |      |         |          |         |
133         NTSVITQ-ACPKVSFEPIPIHYCAPAGFAILKCNNKTFNGTGPCTNVSTVQ
MD          T E SST-T  SS BBB  B  B  TTBBBBBBB  TT  SBBBBSSBBBBS
QMMM        T    SS-S  S  B    B  B  TTBBBBBBB  SS  SBBBBSSBBBBS

               β8     ℒB   β9     ℒC   β10    ℒD    β11      β12
              250        260        270        280        290
               |          |          |          |          |
247         CTHGIRPVVSSQLLLNGSLAEEEVVIRSCNFTDNAKTIIVQLNTSVEINC
MD          B   B SS BSSBBBS    SSS BBB S SS TTS BBBB SS BBBBB
QMMM        B   B SS BSSBBBS    SSS BBB S SS SSS BBBB SS BBBB

                                                   CD4-binding loop
              ℒV3  β13          α2           ℒE   β14     β15    α3
                330        340        350        360        370
                 |          |          |          |          |
297         TGA-GHCNIARAKWNNTLKQIASKLREQFGNNKTIIFKQSSGGDPEIVTHW
MD          BTT-TBBBBBBHHHHHHHHHHHHHHHHHHHH TT EBBB     SSHHHHSBB
QMMM        TTT-SSBBBBBHHHHHHHHHHHHHHHHHHHH TT EBBE     SSHHHHSBB

            β16   β17     α4   β18         ℒV4         β19       β20
               380        390        400        410        420
                |          |          |          |          |
376         FNCGGEFFYCNSTQLFNSTWFNSTWSTEGSNNTEGSDTITLPCRIKQIIN
MD          BBBTTBBBBB  TTTS BBBS SSS TT   S    S SBBBBBBBBBBB B
QMMM        BBBTTBBBBB  HHHS  BBS TTT TTSB      SSBBBB BBBBBB B
                                                   Outer-domain
                                                     exit loop
                   β21    ℒF     β22     β23     ℒV5   β24
               430        440        450        460        470
                |          |          |          |          |
426         MWCKVGKMMYAPPISGQIRCSSNITGLLLTRDGGNSNNESEIFRPGGGDM
MD          BBTTTTBBBBBS TT S   BBBB BBBBBB TT    SSEEEEBB  SST
QMMM        BBTTTTBBBBB    TS   BBBB BBBBBB TT    SSEEEEBB  S T

                 α5       β25
               480        490
                |          |
476         RDNWRSELYKYKVVKI
MD          THHHHHHHTTBBBBB
QMMM        THHHHHHHTTBBBBB
```

Figure S5. gp120 sequence, secondary structure from the crystal structure and the main persistent interacting residues are annotated. Color range for residue contribution to the BFE: $<$ -2.0 kcal/mol (in bold and highlighted in green), contribution range -1.0: -2.0 kcal/mol (highlighted in green), and contribution range -0.25: -1.0 kcal/mol (highlighted in grey). Calculated secondary structure for $\mathbf{I}_{\mathbf{MD}}$ and $\mathbf{I}_{\mathbf{QMMM}}$ set is also shown.

```
                              IgG1 b12 (VH)

                                         H1                H2
                  10        20        30        40        50
                  |         |         |         |         |
1        QVQLVQSGAEVKKPGASVKVSCQASGYRFSNFVIHWVRQAPGQRFEWMGW
MD         BBBB   EEE TT  BBBBBBBBSS GGGS BBBBBB TTS BBB BB
QMMM       BBBB SEEEE TTS BBBBBBBBSS GGGS BBBBBB TTS BBB BB
             H2                                          H3

         52a      60         70      80 82abc      90
          ||       |         |         | ||||       |
51       INPYNGNKEFSAKFQDRVTFTADTSANTAYMELRSLRSADTAVYYCARVG
MD       B TTT  BBB TTTTTTBBBBBBTTTTBBBBBB S  GGG BBBBBBBB
QMMM     B TTT  BBB TTTTTTBBBBBBTTTTBBBBBB S  GGG BBBBBBB
             H3

          100abcdefghij        100       110      120 133
            |||||||||||         |         |         | |
97       PYSWDDSPQDNYYMDVWGKGTTVIVSSASTKGPSVFPLAPSSKS-TSGGTA
MD          TTS TTTT SB BB   BBEEE S   B  BBBBB   GGG-BBTTBB
QMMM        TTS TTTT S  BB   BEEEE     B  BBBBB   STT-BBTTBB

         140       150   156 163   170      180 182    190
          |         |     |  |      |         | |       |
139      ALGCLVKDYFPEPVTV-SW-NSGALTSGVHTFPAVLQS-SGLYSLSSVVTVPS
MD       BBBBBBBSBBSS  BB-BB-GGGTB TTBBB   BB T-TS BBBBBBBBBBG
QMMM     BBBBBBBSBBSS BBB-BB-TTTT  TTBBB   BB T-TS BBBBBBBBBBG

          200 203 205 210      220 225
             | | |    |         |   |
196      SSLGT-Q-TYICNVNHKPSNTKVDKK-AEPKSC
MD       GGTTT-S-  BBBBBBTTTTBBBBBB-
QMMM     GHHTT- -  BBBBBBTTTTBBBBBB-
```

Figure S6. Heavy chains of IgG1 b12 sequence, crystal secondary structure and the main persistent interacting residues. Color range for residue contribution to the BFE: < −2.0 kcal/mol (in bold and highlighted in green), contribution range −1.0: −2.0 kcal/mol (highlighted in green), and contribution range −0.25: −1.0 kcal/mol (highlighted in grey). Calculated secondary structure for $\mathbf{I}_{\mathbf{MD}}$ and $\mathbf{I}_{\mathbf{QMMM}}$ sets are also shown.

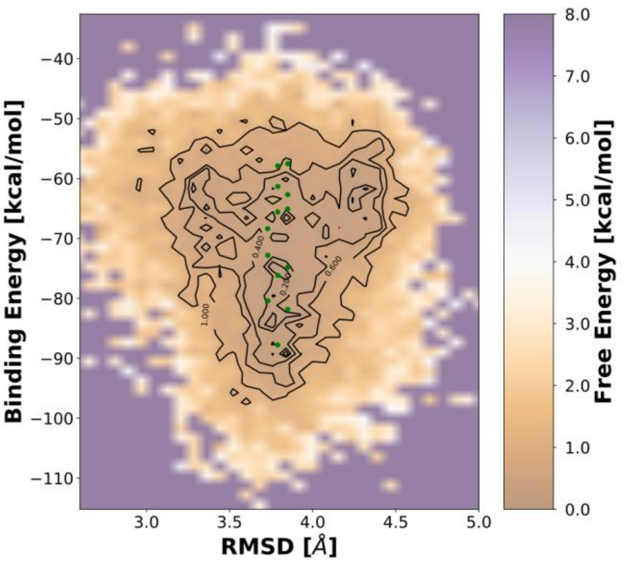

Figure S7. Plot of the two-dimensional potential of mean force (2D-PMF) showing the landscape between the binding free energy and to the root mean square displacement (RMSD) of the b12–gp120 protein-protein complex. The absolute minima (red cross) and starting points of QM/MM MD simulations (green points) are also shown.

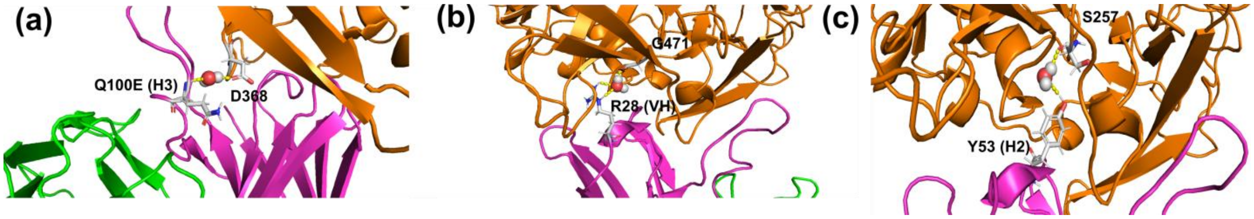

Figure S8. Representative images of relevant water-bridged complexes of the b12−gp120 interface from classical I_{MD} conformational set.

Table S1. Population of the most persistent water-bridged complexes between residues at both sides of the b12–gp120 interface. Sampling obtained from classical MD ($\mathbf{I}_{\mathbf{MD}}$) and QM/MM MD ($\mathbf{I}_{\mathbf{QMMM}}$) trajectories, and from a set of conformations located on the surrounding regions of the absolute minimum of both 2D-PMF profiles, i.e., by correlating variables of binding free energy (BFE) and the RMSD, *f*(BFE,RMSD) $\mathbf{I}_{\mathrm{RMSD}}$, and by correlating the BFE with the buried surface of the interface (BS), *f*(BFE,BS) $\mathbf{I}_{\mathrm{BS}}$.

**B12 Residue**	**GP120 Residue**	$\mathbf{I}_{\mathbf{MD}}$	$\mathbf{I}_{\mathbf{RMSD}}$	$\mathbf{I}_{\mathbf{BS}}$	$\mathbf{I}_{\mathbf{QMMM}}$
Heavy Chain					
N52	D368	55.15%	38.37%	38.02%	48.74%
Q100e	D368	46.8%	30.39%	31.36%	-
R28	G471	39.47%	-	-	-
Y53	S257	30.25%	-	-	-
Y98	G366	-	-	-	37.46%
N54	E370	-	-	-	27.95%

Table S2. Decomposition of the binding free energy on a per-residue basis of the b12 protein of the b12–gp120 complex comparing the set of conformations of the surrounding regions of the absolute minimum of both 2D-PMF profiles, i.e., by correlating variables of binding free energy (BFE) and the RMSD, *f*(BFE,RMSD) $\mathbf{I}_{RMSD}$, and by correlating the BFE with the buried surface of the interface (BS), *f*(BFE,BS) $\mathbf{I}_{BS}$. Only ΔBFE contributions greater than –0.25 kcal/mol are shown. Complementarity determining regions (**CDR**) to which each residue belongs are also shown. Standard deviations are shown in parentheses.

$\mathbf{I}_{RMSD}$			$\mathbf{I}_{BS}$		
		B12			
**Res.**	**CDR**	**ΔBFE**	**Res.**	**CDR**	**ΔBFE**
W100	H3	-11.66(1.91)	W100	H3	-12.15(1.80)
Y98	H3	-5.61(1.02)	Y98	H3	-5.57(0.95)
Y53	H2	-5.56(1.29)	Y53	H2	-5.44(1.03)
F32	H1	-3.37(0.65)	F32	H1	-3.42(0.62)
N31	H1	-3.36(1.24)	N31	H1	-3.04(1.16)
R28	V_H	-2.46(2.07)	R28	V_H	-2.24(1.81)
N100g	H3	-1.56(0.44)	N100g	H3	-1.58(0.44)
P97	H3	-1.53(0.38)	P97	H3	-1.54(0.37)
P100d	H3	-1.04(0.43)	P100d	H3	-1.03(0.46)
Y27	V_H	-0.96(1.90)	N52	H2	-0.81(1.06)
N52	H2	-0.86(1.12)	Y100h	H3	-0.73(0.62)
N56	H2	-0.83(1.40)	V33	H1	-0.72(0.21)
Y100h	H3	-0.79(0.59)	G96	H3	-0.57(0.20)
N54	H2	-0.77(1.64)	V95	H3	-0-54(0.18)
V33	H1	-0.73(0.21)	N54	H2	-0.53(1.38)
G96	H3	-0.57(0.21)	F29	V_H	-0.25(0.11)
V95	H3	-0.54(0.22)			
S74	V_H	-0.45(1.25)			
F59	H2	-0.34(0.47)			
F29	V_H	-0.26(0.14)			

Table S3. Decomposition of the binding free energy on a per-residue basis of gp120 protein for the b12–gp120 complex comparing the set of conformations of the surrounding regions of the absolute minimum of both 2D-PMF profiles, i.e., by correlating variables of the binding free energy (BFE) and the RMSD, *f*(BFE,RMSD) $\mathbf{I}_{RMSD}$, and by correlating the BFE with the buried surface of the interface (BS), *f*(BFE,BS) $\mathbf{I}_{BS}$. Only ΔBFE contributions greater than –0.25 kcal/mol are shown. Standard deviations are shown in parentheses.

$\mathbf{I}_{RMSD}$			$\mathbf{I}_{BS}$	
		GP120		
**Res.**	**ΔBFE[a]**		**Res.**	**ΔBFE[a]**
M475	-3.79(0.74)		M475	-3.92(0.69)
R419	-3.64(1.46)		R419	-3.71(0.57)
I371	-3.36(0.56)		I371	-3.67(0.57)
P369	-3.33(0.49)		P369	-3.32(0.46)
G367	-3.04(0.69)		G367	-3.11(0.66)
G366	-2.74(0.64)		G366	-2.84(0.58)
D474	-2.20(0.78)		D474	-2.18(0.77)
A281	-2.09(1.22)		N386	-1.93(0.51)
N386	-1.89(0.57)		T283	-1.71(2.05)
T455	-1.71(1.72)		A281	-1.66(1.05)
V372	-1.67(0.35)		V372	-1.58(0.35)
G473	-1.56(0.86)		G473	-1.54(0.81)
V430	-1.25(1.58)		T455	-1.50(1.49)
T283	-1.15(1.64)		NAG892	-1.41(1.07)
NAG892	-1.09(0.91)		G471	-0.97(1.28)
S257	-0.96(0.57)		W375	-0.86(0.44)
W375	-0.91(0.46)		S257	-0.81(0.56)
S365	-0.86(0.69)		S365	-0.67(0.75)
G471	-0.70(1.01)		C385	-0.67(0.27)
C385	-0.69(0.34)		T373	-0.66(0.30)
T373	-0.64(0.35)		P417	-0.49(0.23)
G472	-0.64(1.29)		Y384	-0.46(0.25)
P417	-0.52(0.24)		R456	-0.34(1.05)
Y384	-0.42(0.29)		D477	-0.26(0.32)
P470	-0.36(1.11)			
N280	-0.32(1.27)			
G458	-0.29(0.55)			
G431	-0.28(0.61)			
R456	-0.26(0.93)			

Table S4. Binding Energy decomposition on a pairwise per-residue basis for the b12–gp120 protein complex comparing the set of conformations of the surrounding regions of the absolute minimum of both 2D-PMF profiles, i.e., by correlating variables of binding free energy (BFE) and the RMSD, *f*(BFE,RMSD) $\mathbf{I}_{RMSD}$, and by correlating the BFE with the buried surface of the interface (BS), *f*(BFE,BS) $\mathbf{I}_{BS}$. Only contributions lower than –2 kcal/mol are shown. Complementarity determining regions (**CDR**) to which each residue belongs are also shown. Standard deviations are shown in parentheses.

$\mathbf{I}_{RMSD}$					$\mathbf{I}_{BS}$				
**Res.**	**CDR**	**Res.**	**CDR**	**ΔBFE**	**Res.**	**CDR**	**Res.**	**CDR**	**ΔBFE**
W100	H3	R419	GP120	-8.51(2.40)	W100	H3	R419	GP120	-8.85(2.16)
W100	H3	N386	GP120	-4.90(0.87)	W100	H3	N386	GP120	-5.00(0.77)
Y53	H2	M475	GP120	-4.36(0.77)	Y53	H2	M475	GP120	-4.47(0.75)
W100	H3	NAG892	GP120	-3.56(1.93)	W100	H3	NAG892	GP120	-4.14(1.98)
N31	H1	S365	GP120	-3.36(0.92)	Y100h	H3	D368	GP120	-3.19(1.13)
Y100h	H3	D368	GP120	-3.13(1.25)	N31	H1	S365	GP120	-3.14(1.04)
R28	V_H	T455	GP120	-3.13(2.04)	R28	V_H	T455	GP120	-3.09(1.71)
Y98	H3	P369	GP120	-2.96(0.43)	Y98	H3	P369	GP120	-3.02(0.38)
R28	V_H	A281	GP120	-2.79(2.05)	Y53	H2	D474	GP120	-2.69(0.74)
Y53	H2	D474	GP120	-2.77(0.73)	R28	V_H	A281	GP120	-2.66(1.76)
N100g	H3	G367	GP120	-2.43(0.40)	Y53	H2	I371	GP120	-2.49(0.66)
N31	H1	G366	GP120	-2.41(0.34)	N100g	H3	G367	GP120	-2.44(0.40)
E58	H2	K432	GP120	-2.40(3.41)	F32	H1	G366	GP120	-2.41(0.34)
F32	H1	G366	GP120	-2.38(0.37)	N31	H1	G366	GP120	-2.39(0.30)
Y53	H2	I371	GP120	-2.34(0.67)	N31	H1	I371	GP120	-2.27(0.37)
N31	H1	I371	GP120	-2.21(0.39)	S30	V_H	G473	GP120	-2.17(0.72)
S30	V_H	G473	GP120	-2.20(0.70)	Y98	H3	D368	GP120	-2.13(0.41)
Y98	H3	D368	GP120	-2.06(0.49)	R28	V_H	T283	GP120	-2.11(2.39)
N52	H2	D368	GP120	-2.01(1.46)	Y98	H3	G366	GP120	-2.02(0.32)
Y98	H3	G366	GP120	-2.01(0.35)					
